# Prevalence and Determinants of Diabetes and Pre-diabetes Among Adults in a Rural Region in India: A Community-Based Cross-Sectional Study

**DOI:** 10.7759/cureus.117925

**Published:** 2026-10-14

**Authors:** Gudluru Uday Sai, Praveen Kumar BA, Devi Varaprasad, Janakiraman Pichandi, Sanju N Raju

**Affiliations:** 1 Community Medicine, Sri Venkateswaraa Institute of Medical College Hospital and Research Institute, Chennai, IND; 2 Community Medicine, PES Institute of Medical Sciences and Research, Kuppam, IND; 3 Community Medicine, Andhra Medical College, Visakhapatnam, IND; 4 Biostatistics, PES Institute of Medical Sciences and Research, Kuppam, IND

**Keywords:** andhra pradesh, diabetes mellitus, prevalence, risk factors, rural health

## Abstract

Background and objective

Diabetes mellitus (DM) and pre-diabetes are growing public health concerns in rural Indian communities. Local occupational patterns, dietary habits, and socioeconomic factors often shape dysglycemia risk profiles in these areas. Consequently, this study was conducted to estimate the prevalence of diabetes and pre-diabetes and identify the key sociodemographic, behavioral, and anthropometric determinants of these conditions among adults in the rural setting of Andhra Pradesh. The study aimed to estimate the prevalence of diabetes and pre-diabetes among adults aged 35 years and above and to determine the sociodemographic, behavioral, and anthropometric factors associated with these conditions.

Methods

This community-based cross-sectional study was conducted between March 2021 and February 2022 among 450 adults aged 35 years and above in rural Kuppam Mandal. A multistage sampling strategy was employed, with nine village clusters selected through simple random sampling and 50 households per cluster selected using systematic random sampling. We measured fasting blood glucose (FBG) levels and categorized them according to the American Diabetes Association (ADA) criteria. We collected data on sociodemographic factors, lifestyle behaviors, family history, and anthropometric measurements using structured interviews and standardized physical examinations. Bivariate chi-square tests and multivariable logistic regression analyses were performed to identify independent determinants of dysglycemia, with statistical significance set at p < 0.05.

Results

The overall prevalence of hyperglycemic status was 39.8%, including 20.7% diabetes and 19.1% pre-diabetes. Significant independent associated factors included age ≥ 50 years (adjusted odds ratio (AOR) = 3.4), higher socioeconomic status (SES) (AOR = 1.6), positive family history of diabetes (AOR = 2.1), alcohol consumption (AOR = 3.2), BMI > 25 kg/m² (AOR = 2.4), and comorbidities (AOR = 3.2).

Conclusions

We found a substantial burden of diabetes and pre-diabetes in the rural setting, with a significant association with age, alcohol consumption, and the presence of comorbidities, highlighting the vital role of public health professionals and policymakers in implementing targeted interventions for better health outcomes.

## Introduction

Diabetes mellitus (DM) is a metabolic disorder characterized by persistent hyperglycemia resulting from defects in insulin secretion, insulin action, or both. Under physiological conditions, insulin maintains glucose homeostasis by facilitating cellular glucose uptake and suppressing hepatic glucose output; impairment in these mechanisms leads to sustained elevations in blood glucose levels [1]. Pre-diabetes is an intermediate metabolic state characterized by glycemic levels above normal physiological thresholds yet below the diagnostic cutoff for overt diabetes [2].

DM and pre-diabetes have become significant global health challenges, affecting populations in both developed and developing countries. In India, urban areas have experienced a rapid increase in diabetes and pre-diabetes over the past decade. In contrast, rural communities have traditionally been considered less susceptible to these non-communicable diseases due to physically active lifestyles and diets rich in unprocessed foods. However, ongoing lifestyle changes and the increasing availability of processed foods have gradually eroded this protective effect. Additionally, the cultural acceptance of risk behaviors, including tobacco use and alcohol consumption, has further contributed to the growing prevalence of these conditions [3-5].

This epidemiological shift is particularly concerning given the unique challenges faced by rural populations, including limited access to healthcare, low awareness of diabetes, and socioeconomic constraints [6]. Among adults aged 35 years and above, these factors may further increase the burden of diabetes and pre-diabetes and the risk of severe complications, such as cardiovascular disease, kidney failure, and visual impairment, if these conditions remain undiagnosed or untreated [7].

Despite the critical need for reliable data, epidemiological studies among rural Indian populations remain scarce, limiting our understanding of rural-specific determinants, such as occupational patterns, educational status, and household structures, that may influence disease onset and progression [3-7]. A previous house-to-house survey conducted in Kuppam in 2017 identified 1,339 self-reported cases of diabetes, corresponding to a prevalence of 6.8% among 19,654 adults. However, diabetes identification was based primarily on self-reported illness, medication use, and available laboratory records, highlighting the need for population-based assessments using objective glycemic measurements [8]. To address this knowledge gap, the present study was conducted in rural Kuppam to estimate the prevalence of diabetes and pre-diabetes and identify the factors associated with these conditions among adults aged 35 years and above.

## Materials and methods

This community-based cross-sectional epidemiological study was conducted for a period of 12 months from March 2021 to February 2022 among adults aged 35 years and above in the rural area of a southern state of India

Sampling technique and sample size

To ensure a representative sample of the rural population in Kuppam Mandal, a multistage sampling approach was employed. Kuppam Mandal consists of 63 revenue villages/panchayats. In the first stage, villages with at least 400 households were identified to establish an adequate sampling frame, from which nine villages were selected using simple random sampling to serve as study clusters. In the second stage, 50 households were selected from each of the nine villages, yielding a total sample of 450 households. Within each village, spatial randomization was performed by using the last digit of a currency note to select the starting area or street and a coin toss to determine the side of the road. From this starting point, systematic random sampling was used to select every alternate household. Finally, within each selected household, eligible individuals aged 35 years and above who provided informed consent were enrolled in the study. When more than one eligible individual was present in a household, one individual was randomly selected for participation (Figure 1).

**Figure 1:**
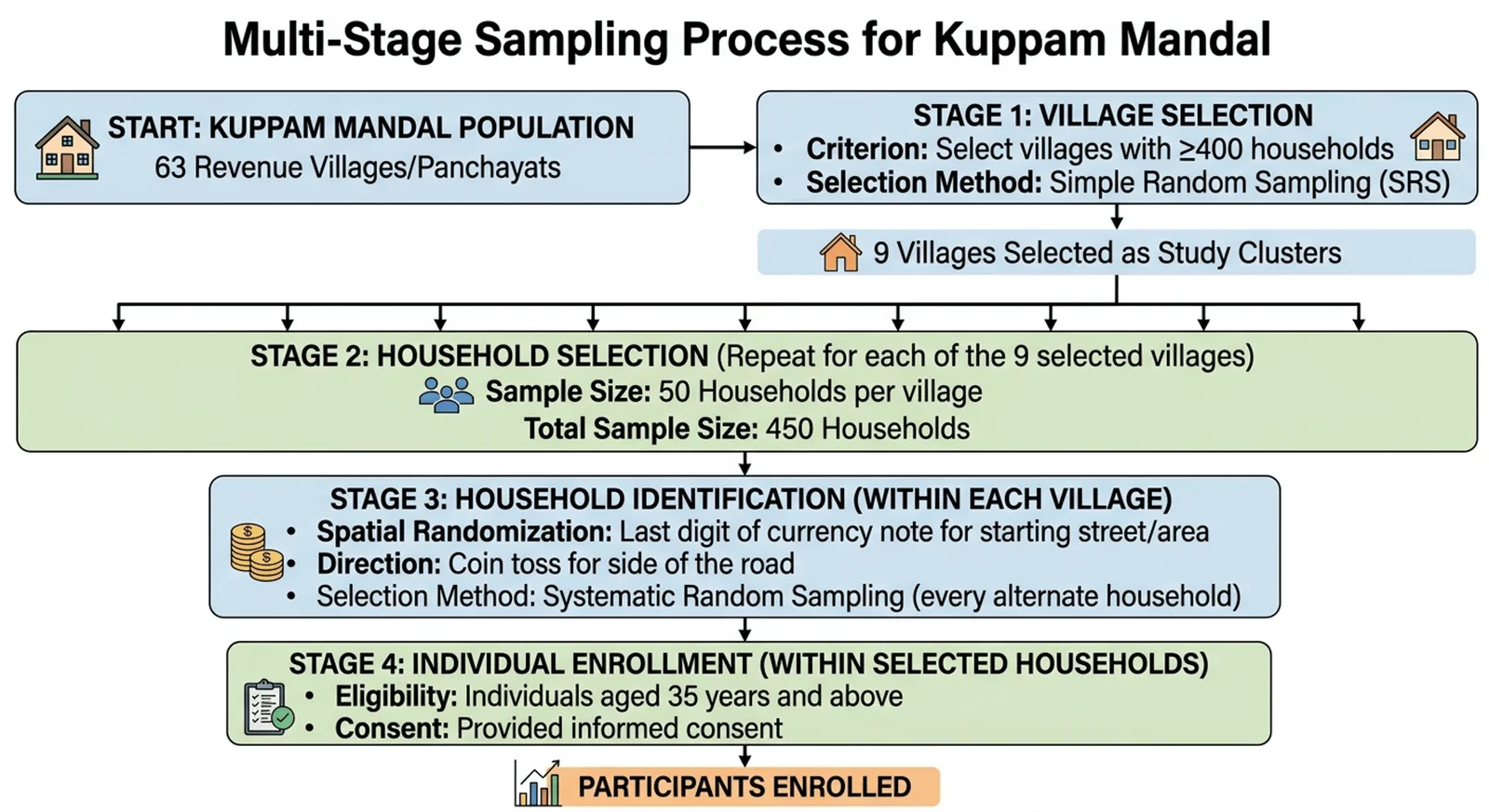
Flowchart illustrating the multistage sampling strategy, from regional village cluster selection to household inclusion criteria Image generated using Google Gemini (Alphabet Inc., Mountain View, CA)

The sample size was derived using the following formula:

$n = \frac{4p(1-p)}{L^2}$

where ‘p’ was 27.8%, based on the combined prevalence of diabetes and pre-diabetes reported by Prabhune et al. [9], and the allowable relative error, ‘L’, was set at 4.448% of ‘p’, with a 95% confidence interval (CI). This yielded an initial sample size of 406. After accounting for a 10% non-response rate, the final estimated sample size was 447, which was rounded up to 450 to facilitate equal distribution across clusters.

Data collection

A self-developed, structured, and pre-validated questionnaire underwent face and content validation and was translated into Telugu and back-translated to ensure linguistic and conceptual equivalence before being used for data collection. Anthropometric measurements, including weight (kg) and height (cm), were obtained using calibrated weighing scales and stadiometers. Fasting blood glucose (FBG) was measured using capillary blood samples collected after an 8-12-hour fast, with a calibrated glucometer (Accu-Chek Instant, Model 929; Roche Diagnostics, Rotkreuz, Switzerland).

Participants were instructed on fasting compliance the previous day, and follow-up visits were scheduled to maximize data completeness. FBG was measured after an overnight fast of at least eight hours. Skin at the puncture site (typically the side of the fingertip) was cleansed with an alcohol swab and allowed to air-dry completely before obtaining a drop of capillary blood via a single-use sterile lancet. However, participants with elevated capillary blood glucose readings (> 126 mg/dL for fasting or > 200 mg/dL for random) underwent an immediate repeat capillary test using a fresh test strip. People with diabetes on treatment were classified as diabetic regardless of current FBG values.

Glycemic categories were based on the American Diabetes Association (ADA) 2021 criteria [10]: normal (< 100 mg/dL), pre-diabetes (100-125 mg/dL), diabetes (≥ 126 mg/dL or prior diagnosis under treatment) (Table 1).

**Table 1:** American Diabetes Association (ADA) 2021 criteria (fasting plasma glucose level)

Category	Values
Normal	< 100 mg/dL
Pre-diabetes	100–125 mg/dL
Diabetes	≥ 126 mg/dL or prior diagnosis under treatment

Furthermore, physical activity was assessed using self-reported occupational profiles and qualitative questions regarding regular exercise (such as daily walking) rather than a validated quantitative instrument like the International Physical Activity Questionnaire (IPAQ).

Ethical considerations

Approval from the Institutional Human Ethics Committee (PESIMSR/IHEC/47-21) at PES Institute of Medical Sciences and Research, Kuppam was obtained. Written informed consent was obtained from each participant before inclusion. Newly identified individuals with elevated glucose levels were counseled and referred to appropriate healthcare facilities. Educational interventions regarding diabetes prevention and control were uniformly provided.

Data analysis

Data were entered into Microsoft Excel 2019 [11] and analyzed using IBM SPSS Statistics version 21 (IBM Corp., Armonk, NY) [12]. Descriptive statistics were used to summarize demographic and clinical characteristics. Inferential analyses included chi-square tests for categorical variables and multivariable logistic regression to adjust for potential confounders and identify factors associated with dysglycemia. The reference categories were age < 50 years, socioeconomic status (SES) classes I and II, no family history of diabetes, no tobacco consumption, BMI < 25 kg/m², and no history of comorbidities. Statistical significance was set at p < 0.05.

## Results

A total of 450 adults aged 35 years and above from rural Kuppam Mandal, Andhra Pradesh, participated in the study. The mean age of the participants was 51.8 ± 4.9 years, with the highest proportion belonging to the age group below 50 years (321, 71.3%). Male participants accounted for 244 (54.2%) of the sample. Most participants were literate (396, 88%), were engaged in occupations classified as “other” (306, 68%), and lived in nuclear families (371, 82.4%). Most participants (275, 61.1%) belonged to socioeconomic classes IV/V, while the remaining 175 (38.9%) belonged to classes I/II/III, according to the Modified BG Prasad classification [13], updated in February 2021. A positive family history of diabetes was reported by 98 (21.8%) participants. Risk behaviors included tobacco use in 75 (16.7%) participants and alcohol consumption in 68 (15.1%). Overweight and obesity (BMI > 25 kg/m²) were observed in 199 (44.2%) participants. Moderate-intensity physical activity was reported by 313 (69.6%) participants. Other comorbid conditions were reported by 41 (9.1%) participants (Table 2).

**Table 2:** Sociodemographic, behavioral, and clinical characteristics of the study participants (n = 450) SES: socioeconomic status; H/O: history; BMI: body mass index

Variable		Total	
		N	%
Age, years	< 50	321	71.3
	> 50	129	28.7
Gender	Male	244	54.2
	Female	206	45.8
Education	Illiterate	54	12.0
	Literate	396	88.0
Occupation	Business/employee	144	32.0
	Others	306	68.0
Family	Nuclear	371	82.4
SES	Class I/II/III	175	38.9
	Class IV/V	275	61.1
Family H/O	Present	98	21.8
	Absent	352	78.2
Tobacco	Used	75	16.7
	Not used	375	83.3
Alcohol	Consumed	68	15.1
	Not consumed	382	84.9
BMI, kg/m^2^	< 25	251	55.8
	> 25	199	44.2
Activity	Moderate intensity	313	69.6
	Others	137	30.4
Comorbidity	Present	41	9.1
	Absent	409	90.9

Based on FBG levels, the participants were classified as having normal glucose levels, pre-diabetes, or diabetes. Participants with a known history of diabetes were classified as diabetic, irrespective of their FBG levels. Accordingly, 271 (60.2%) participants had normal glucose levels, 86 (19.1%) had pre-diabetes, and 93 (20.7%) had diabetes. The overall prevalence of hyperglycemia among the study participants was 179 (39.8%) (Figure 2).

**Figure 2:**
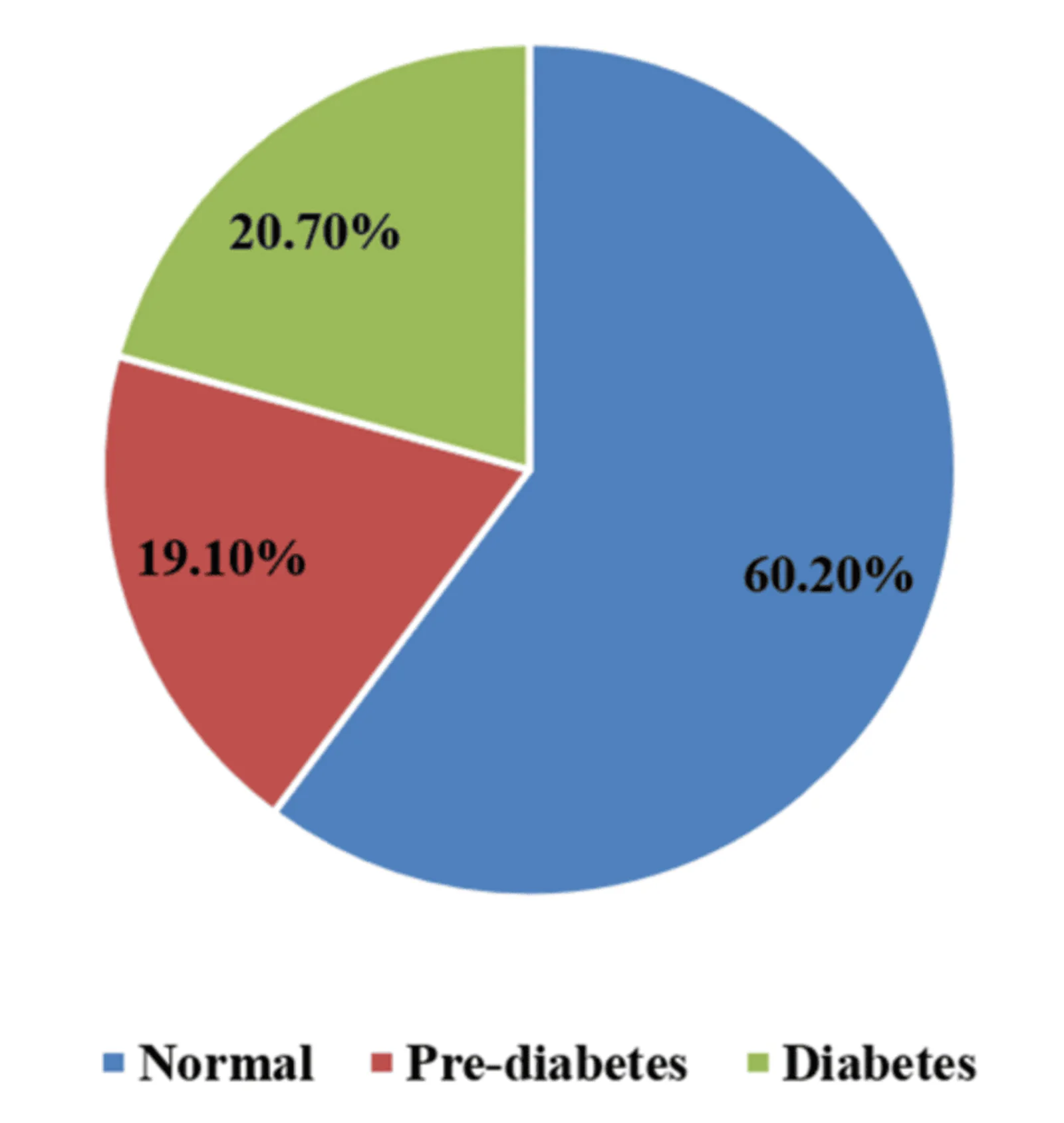
Prevalence of pre-diabetes and diabetes among the study subjects (n = 450)

The association of hyperglycemic status with non-modifiable and modifiable factors showed that the occurrence of pre-diabetes/diabetes was significantly associated with increasing age (χ² = 42.71, p < 0.001), higher socioeconomic status (χ² = 6.21, p < 0.013), family history of diabetes (χ² = 13.97, p < 0.001), tobacco use (χ² = 11.58, p < 0.001), alcohol consumption (χ² = 20.78, p < 0.001), BMI > 25 kg/m² (χ² = 29.15, p < 0.001), and the presence of comorbidities (χ² = 24.18, p < 0.001). However, although variables such as male sex, illiteracy, occupations involving potentially lower physical activity (such as owning a shop/business or salaried employment), living in a non-nuclear family, and self-reported moderate-intensity physical activity were associated with a higher prevalence of pre-diabetes/diabetes, these associations were not statistically significant (Table 3).

**Table 3:** Factors associated with pre-diabetes and diabetes mellitus among the study subjects (n = 450) ^*^Statistically significant SES: socioeconomic status; H/O: history; BMI: body mass index

Variable		Pre-diabetes/diabetes				Total		Degrees of freedom (df)	χ² value (p-value)	Effect size (ϕ)
		Present (N = 179)		Absent (N = 271)						
		N	%	N	%	N	%			
Age, years	< 50	97	30.2	224	69.8	321	71.3	1	42.71 (< 0.001)^*^	0.308
	> 50	82	63.6	47	36.4	129	28.7			
Gender	Male	103	42.2	141	57.8	244	54.2	1	1.32 (0.251)	0.054
	Female	76	36.8	130	63.2	206	45.8			
Education	Illiterate	27	50	27	50	54	12	1	2.68 (0.102)	0.077
	Literate	152	38.4	244	61.6	396	88			
Occupation	Business/employee	61	42.4	83	57.6	144	32	1	0.59 (0.442)	0.036
	Others	118	38.6	188	61.4	306	68			
Family	Nuclear	141	38.1	230	61.9	371	82.4	1	2.42 (0.120)	0.073
	Others	38	47.5	41	52.5	79	17.6			
SES	Class I/II/III	57	32.5	118	67.4	175	38.9	1	6.21 (0.013)^*^	0.118
	Class IV/V	122	44.4	153	55.6	275	61.1			
Family H/O	Present	55	56.1	43	43.9	98	21.8	1	13.97 (< 0.001)^*^	0.176
	Absent	124	35.2	228	64.8	352	78.2			
Tobacco	Used	43	57.3	32	42.7	75	16.7	1	11.58 (< 0.001)^*^	0.160
	Not used	136	36.3	239	63.7	375	83.3			
Alcohol	Consumed	44	64.7	24	35.3	68	15.1	1	20.78 (< 0.001)^*^	0.215
	Not consumed	135	35.3	247	64.7	382	84.9			
BMI, kg/m^2^	< 25	72	28.7	179	71.3	251	55.8	1	29.15 (< 0.001)^*^	0.255
	> 25	107	53.8	92	46.2	199	44.2			
Activity	Moderate intensity	116	37.1	197	62.9	313	69.6	1	3.16 (0.075)	0.084
	Others	63	45.9	74	54.1	137	30.4			
Comorbidity	Present	31	75.6	10	24.4	41	9.1	1	24.18 (< 0.001)^*^	0.232
	Absent	148	36.2	261	63.8	409	90.9			

Multivariable logistic regression analysis identified factors independently associated with dysglycemia (pre-diabetes and diabetes combined) among adults aged 35 years and older in rural Kuppam Mandal. Participants aged 50 years and above had threefold higher odds of dysglycemia. Alcohol consumption, the presence of comorbidities, elevated BMI (> 25 kg/m²), a positive family history of diabetes, and higher socioeconomic status (classes IV/V) were also independently associated with dysglycemia. In contrast, tobacco use was no longer statistically significant after adjustment for potential confounders in the multivariable model (Table 4).

**Table 4:** Multiple logistic regression analysis of the determinants of pre-diabetes and diabetes among the study subjects (n = 450) ^*^Statistically significant AOR: adjusted odds ratio; CI: confidence interval; SES: socioeconomic status; H/O: history; BMI: body mass index

Independent variable	Beta	Standard error (SE)	Wald	Degrees of freedom (df)	AOR	95% CI	P-value
								Age less than 50 years					1	(2.1-5.5)	0
Age 50 years and above	1.2	0.8	24.9	1	3.4										
SES Class I/II/III					1	(1-2.5)	0.036^*^
SES Class IV/V	0.4	0.4	4.4	1	1.6		
No family H/O diabetes					1	(1.2-3.6)	0.004^*^
Positive family H/O diabetes	0.7	0.6	8.2	1	2.1		
No tobacco usage					1	(0.6-2.4)	0.445
Tobacco usage	0.1	0.4	0.5	1	1.2		
No H/O alcohol consumption					1	(1.6-6.2)	0.001^*^
Alcohol consumption	1.1	1.1	11.6	1	3.2		
BMI < 25 kg/m^2^					1	(1.5-3.7)	0.001^*^
								BMI > 25 kg/m^2^	0.9	0.53	15	1	2.4
No H/O of comorbidities					1									(1.4-7.3)	0.005^*^
								Presence of comorbidities	1.1	1.3	7.6	1	3.2		

## Discussion

Diabetes mellitus and pre-diabetes represent some of the most profound and urgent global health challenges of our time, transcending geographic, socioeconomic, and cultural boundaries. The observed prevalence highlights that dysglycemia is a major public health concern in both rural and urban settings. Ramachandran et al. [14] and Chandrupatla et al. [15] similarly described an expanding diabetes epidemic extending into rural communities, with comparable magnitude and complexity. Our findings align with the growing epidemiological evidence that diabetes is increasingly affecting diverse demographic groups and settings. As Ramachandran et al. [14] and Chandrupatla et al. [15] noted, this rising tide imposes severe burdens on individuals, families, and healthcare systems. Addressing this challenge requires global and national health agendas to urgently prioritize rural vulnerabilities alongside urban health challenges [14,15].

An earlier community-based survey in Kuppam, reported by Bharani AR et al. [8], found a 6.8% prevalence of self-reported diabetes among adults aged 18 years and above in 2017. The present study, conducted among adults aged 35 years and above, used fasting capillary blood glucose assessment and identified a prevalence of diabetes (20.70%) and pre-diabetes (19.10%). Differences in age eligibility and case ascertainment preclude direct comparison of prevalence estimates, but the findings underscore the importance of objective glycemic screening in rural populations.

Age emerged as an important non-modifiable risk factor in this population, with individuals aged 50 years and older demonstrating approximately threefold higher odds of dysglycemia, according to the multivariable analysis. This observation aligns with previous studies by Tabák et al. [2] and Wang et al. [16], reinforcing the strong association between advancing age and impaired glucose regulation. Physiologically, ageing may increase vulnerability to progressive metabolic dysfunction through age-related declines in pancreatic beta-cell function, increased insulin resistance, and prolonged exposure to unfavorable lifestyle risk factors.

Identifying modifiable risk factors offers a promising basis for public health intervention in rural populations. Notably, elevated BMI showed a robust positive association with dysglycemia, strongly corroborating previous observations by Prabhune et al. [9], Goel et al. [17], and Gupta et al. [18]. From a pathophysiological perspective, excess adiposity, particularly visceral fat accumulation, can trigger chronic low-grade systemic inflammation, increase circulating free fatty acids, and disrupt normal insulin signalling pathways. These mechanisms can promote peripheral insulin resistance and may help explain the association observed in the present study.

A positive family history of diabetes was associated with an increased likelihood of dysglycemia, highlighting the interplay of inherited polygenic susceptibility and shared environmental influences. Genetic susceptibility may affect pancreatic beta-cell function and peripheral insulin sensitivity, while shared dietary habits and lifestyle patterns may further contribute to risk. These findings are consistent with those reported by Anjana et al. [19] and Barik et al. [20]. In this context, a positive family history reflects the combined influence of a shared familial environment, behavioral patterns, and potential genetic factors rather than isolated genetic susceptibility. Furthermore, tobacco and alcohol use may contribute additional metabolic risks, consistent with the findings of Barik et al. [20] and Okwechime et al. [21]. Mechanistically, tobacco use can induce systemic oxidative stress and endothelial dysfunction, while chronic alcohol consumption may disrupt hepatic glucose metabolism and pancreatic endocrine function. Ultimately, while family history indicates baseline vulnerability, these findings suggest that family history and potentially modifiable behavioral factors are associated with dysglycemia and may help identify individuals who could benefit from targeted screening.

Notably, gender was not a significant independent predictor of dysglycemia in this cohort, aligning with the findings of Goel et al. [17] but diverging from those of Hariri et al. [23]. The absence of a statistically significant independent association with gender may reflect the influence of shared environmental and lifestyle factors in this rural setting. Sociocultural dynamics unique to rural regions, including potentially similar occupational demands among men and women, may also contribute to overlapping risk profiles. However, these explanations remain hypothetical and require further investigation. The findings support inclusive, population-wide preventive strategies rather than interventions based solely on gender.

Socioeconomic status (SES) exhibited a relationship with dysglycemia that may reflect broader socioeconomic and epidemiological transitions, consistent with trends observed by Talukder et al. [23] and Patil et al. [24]. In rapidly developing rural regions, higher socioeconomic status may facilitate dietary transitions characterized by increased consumption of energy-dense, processed foods and reduced occupational physical activity due to mechanization and improved transportation. These changes may contribute to an obesogenic environment and increase metabolic disease risk across socioeconomic strata.

Collectively, these findings underscore the urgent need for culturally appropriate, community-based interventions emphasizing early detection, lifestyle modification, and behavioral risk reduction in rural settings [19,25]. Evidence from a related community-based quasi-experimental study in South India further supports the role of structured health education in diabetes care. In that study, small-group health education was associated with significant improvements in self-care practices and a reduction in mean fasting blood glucose among adults with type 2 diabetes [26].

The findings of the present study highlight diabetes and pre-diabetes as major public health concerns with multifactorial determinants, including age, adiposity, family history, and lifestyle-related factors. These challenges extend across rural and urban settings and call for coordinated national and international action. Multisectoral approaches, ranging from grassroots community engagement to policy-level interventions, are needed to address the growing burden of dysglycemia [27,28].

Recommendations

Community-based screening strategies should be implemented to identify dysglycemia early, with particular emphasis on high-risk rural populations. Such approaches could facilitate timely diagnosis and appropriate clinical management. Culturally appropriate and context-specific lifestyle modification programmes should also be developed and implemented, emphasizing healthy dietary practices, increased physical activity, and the reduction of harmful behavioral risk factors. These programmes should be tailored to local dietary practices, cultural preferences, and the socioeconomic circumstances of rural communities.

Limitations

Behavioral and lifestyle determinants were assessed through questionnaire-based interviews, which provided comprehensive data; however, inherent recall and reporting variability should be acknowledged. The use of fasting capillary blood glucose measurements obtained with a glucometer, without confirmatory laboratory testing, may have affected the accuracy of glycemic classification. Furthermore, physical activity was assessed using self-reported occupational profiles and qualitative questions about regular exercise, such as daily walking, rather than a validated quantitative instrument such as the IPAQ. This approach may have introduced reporting and classification bias.

The cross-sectional design precludes establishing temporal or causal relationships between the identified factors and dysglycemia. Although complex survey procedures were applied to adjust standard errors for the sampling design, residual effects of clustering at the village or household level may still have influenced the precision of the estimates. Potential selection bias may also have affected the findings. While these results reflect epidemiological patterns observed in rural South India, they may not be directly generalisable to urban cohorts, tribal populations, or geographically diverse rural regions of northern and eastern India, where dietary, socioeconomic, and cultural environments differ substantially. Therefore, the generalizability of the findings to these populations is limited.

## Conclusions

The study findings indicate that the ongoing epidemiological transition in rural South India is influenced by a synergistic combination of non-modifiable factors, such as age and family history, and modifiable lifestyle-related risk factors. Rather than relying on resource-intensive universal screening, rural health system strategies should prioritize structured, community-based risk stratification and opportunistic screening among high-risk groups, specifically older adults, individuals with an elevated BMI (> 25 kg/m²), those with a positive family history of diabetes, and individuals with pre-existing chronic comorbidities. Furthermore, preventive public health initiatives should integrate culturally adapted behavioral interventions, emphasizing community-level weight management and targeted interventions to reduce alcohol-related harm, to effectively mitigate the growing burden of dysglycemia in rural populations.

## Appendices

Proforma

Title: Prevalence and factors associated with diabetes and pre-diabetes among adults aged 35 years and above in a rural community of Andhra Pradesh

-----------------------------------------------------------------------------------------------------------

Date of visit: ……………; Identification number of participant: ………..

Section I: Sociodemographic Details of the Study Participant

Name:

Address: House No: …………….; Street name:…………………………; Village: ……………………; Mobile number: ………………....

1) Gender: Male/Female

2) Age (in completed years): ……………......years

3) Religion: Hindu/Muslim/Christian/Others (specify): ………………....

4) Marital status: Not married/married/separated/divorced/widowed

5) Educational status: Illiterate/Primary school/Secondary school/College

6) Occupation: ………………......

7) Type of family: Nuclear/Joint/Three generations

8) Total members of the family: ………………......

9) Total income of the family per month: Rs: ………………......

-----------------------------------------------------------------------------------------------------------

Section II: Family History of Diabetes

10) Do you have any family history of diabetes? Yes/No

10a) If yes, specify the relationship of that patient to you: ……………......

-----------------------------------------------------------------------------------------------------------

Section III: Dietary Habits

11) Do you eat junk food? (E.g., Oily or fatty food/Fried food): Yes/No

11. a) If yes, how often? Daily/Frequently/Occasionally

11. b) If yes, how much each time? ………………......

12) In a typical week, on how many days do you eat vegetables? ………

12. a) How many servings in a day? ………………......

13) In a typical week, on how many days do you eat fruits? ………………......

13. a) How many servings in a day? ……………….......

-----------------------------------------------------------------------------------------------------------

Section IV: Behavior

Tobacco usage:

14) Do you use tobacco in any form (smoking/chewing/sniffing)? Never used/currently using/previously used and now stopped using

14a) If currently using, how many times per day do you use? ………………......

14b) If previously used, how many years did you use tobacco? ………………...

Alcohol consumption

15) Do you consume/use alcohol? Never used/currently using/previously used and now stopped using

15a) If currently using, how often? Daily/weekends/occasionally

15b) If previously consumed, how many years did you use alcohol? .............

15c) If previously used, how often? Daily/weekends/occasionally

-----------------------------------------------------------------------------------------------------------

Section V: Physical Activity/Exercise

Physical activity (physical work)

16) In your occupation, what type of physical activity are you involved in most of the time? Mild intensity/moderate intensity/vigorous intensity

Exercise

17) Do you perform any exercise regularly? Yes/No

17a) If yes, what is the duration of that exercise per day? ………………......

17b) If yes, how many days per week? ……………….....

-----------------------------------------------------------------------------------------------------------

Section VI: Known Morbidities

18) Are you a known case of diabetes? Yes/No

18a) If yes, do you take the medications regularly? Yes/No

19) Are you a known case of any other chronic disease? Yes/No

19a) If yes, what is it? Hypertension/kidney disease/any other (specify) ……………………

-----------------------------------------------------------------------------------------------------------

Measurements

Anthropometric measurements:

20a) Weight: ……………….....kg

20b) Height:……………….....meter

Blood glucose measurement

21) Fasting blood sugar (FBS): …….………………….....

*****
